# Survival of immunobiological drugs in psoriasis: preliminary data from a Tertiary Hospital experience in Southern Brazil^☆^^☆☆^

**DOI:** 10.1016/j.abd.2020.08.011

**Published:** 2021-03-15

**Authors:** Elis Costa de Lima, Juliana Catucci Boza, Penélope Esther Palominos, Ricardo Machado Xavier, Tania Ferreira Cestari

**Affiliations:** a Department of Dermatology, Hospital de Clínicas de Porto Alegre, Universidade Federal do Rio Grande do Sul, Porto Alegre, RS, Brazil; b Department of Rheumatology, Hospital de Clínicas de Porto Alegre, Universidade Federal do Rio Grande do Sul, Porto Alegre, RS, Brazil

Dear Editor,

The development of immunobiological drugs has had a major impact on the treatment of psoriasis. Drug survival is the time period between the beginning of treatment and its interruption.^1^ Since the main reason for drug discontinuation is the loss of efficacy, drug survival can be considered a measure of the likelihood of success for biological drugs in patients with moderate to severe psoriasis.^2^ Studies that assess the survival of immunobiological drugs in the population of Brazil are necessary.

This is a retrospective cohort that included patients with psoriasis who used biological drugs, at the Dermatology Service of the Hospital das Clínicas de Porto Alegre, from June 2007 to March 2018. Electronic medical records were reviewed. The inclusion criteria were: patients who started using Adalimumab (ADA), Infliximab (IFX), Etanercept (ETN), Ustekinumab (UST), or Secukinumab (SEC), either as the first, second, third, or fourth line of biological treatment in the observed period. Ixekizumab, Guselkumab, and Risankizumab were not included in the analysis as they were not yet available in Brazil. The treatment courses were considered individually, patients who received more than one immunobiological during their participation in thestudy were included more than once in the analysis, considering each biological drug or treatment line as a new inclusion. Patients who did not discontinue treatment until the study was completed were censored.

Statistical analyses included treatment discontinuation for any cause and analyses were also carried out censoring interruptions as a result of causes not directly associated with treatment efficacy and tolerance (patient's wish, pregnancy plan, interruption of drug supply). Drug survival was analyzed using the Kaplan-Meier curves and was compared using the Log Rank test. The risk factors for discontinuation were identified by Cox regression. For all tests, the significance level was set at 0.05.

A total of 106 courses of treatment with ADA (n = 36), ETN (n = 13), IFX (n = 8), UST (n = 36), and SEC (n = 13) were administered to 75 patients with moderate to severe psoriasis, and the patients characteristics are shown in Table 1. Twenty-one patients (28.0%) used more than one course of treatment.

**Table 1:** Characteristics of psoriasis patients treated with immunobiological drugs – first line of treatment.

	Drug					
	ADA	ETN	IFX	UST	SEC	Total
N. of patients (% of total)	30 (40.0)	10 (13.3)	2 (2.6)	26 (43.6)	7 (9.3)	75 (100)
N. of Men/Women (% of men)	19 / 11 (63.3)	5 / 5 (50.0)	1 / 1 (50.0)	17 / 9 (65.4)	5 / 2 (71.4)	47 / 28 (62.7)
Age in years (mean ± SD)	53.7 (11.2)	51.2 (16.1)	52.5 (17.6)	45.9 (12.7)	48.8 (12.2)	50.2 (12.8)
Duration of disease^a^ (mean ± SD)	19.5 (8.6)	26.3 (17.3)	15.5 (3.5)	18.6 (8.4)	16.8 (6.7)	19.7 (9.9)
Socioeconomic level n (%)						
Low	28 (93.3)	9 (90.0)	2 (100)	25 (96.2)	2 (28.6)	66 (88.0)
Intermediate	2 (6.7)	1 (10.0)	0 (0.0)	1 (3.8)	5 (71.4)	9 (12.0)
N. of comorbidities, n (%)						
0	1 (3.3)	3 (30.0)	0 (0.0)	5 (19.2)	1 (14.3)	10 (13.3)
1	9 (30.0)	2 (20.0)	0 (0.0)	12 (46.2)	4 (57.1)	27 (36.0)
2	6 (20.0)	1 (10.0)	0 (0.0)	4 (15.4)	2 (28.6)	13 (17.3)
≥ 3	14 (46.7)	4 (40.0)	0 (0.0)	5 (19.2)	0 (0.0)	25 (33.3)
Psoriatic arthritis, n (%)	24 (80.0)	5 (50.0)	2 (100)	3 (11.5)	2 (28.6)	36 (48.0)
Obesity, n (%)	7 (23.3)	2 (20.0)	2 (100)	8 (30.8)	1 (14.3)	20 (26.7)
Active smoking, n (%)	5 (16.7)	1 (10.0)	0 (0.0)	3 (11.5)	1 (14.3)	10 (13.3)
Diabetes mellitus, n (%)	8 (26.7)	2 (20.0)	1 (50.0)	8 (30.8)	1 (14.3)	20 (26.7)
Depression, n (%)	7 (23.3)	3 (30.0)	0 (0.0)	3 (11.5)	1 (14.3)	14 (18.7)
Hypertension, n (%)	12 (40.0)	4 (40.0)	1 (50.0)	8 (30.8)	2 (28.6)	27 (36.0)
Dyslipidemia, n (%)	9 (30.0)	1 (10.0)	1 (50.0)	3 (11.5)	0 (0.0)	14 (18.7)
CVD, n (%)	3 (10.0)	1 (10.0)	0 (0.0)	0 (0.0)	0 (0.0)	4 (5.3)
Previous Treatments^b^, n (%)						
0	0 (0.0)	0 (0.0)	0 (0.0)	1 (3.8)	0 (0.0)	1 (1.3)
1	16 (53.3)	1 (10.0)	2 (100)	1 (3.8)	0 (0.0)	20 (26.6)
2	7 (23.3)	3 (30.0)	0 (0.0)	13 (50.0)	2 (28.6)	25 (33.3)
3	2 (6.7)	6 (60.0)	0 (0.0)	8 (30.8)	1 (14.3)	17 (22.6)
4	5 (16.7)	0 (0.0)	0 (0.0)	3 (11.5)	3 (42.9)	11 (14.6)
5	0 (0.0)	0 (0.0)	0 (0.0)	0 (0.0)	1 (14.3)	1 (1.3)
Methotrexate^c^, n (%)	18 (60.0)	4 (40.0)	0 (0.0)	3 (11.5)	0 (0.0)	25 (33.3)
Cyclosporine^c^, n (%)	0 (0.0)	0 (0.0)	0 (0.0)	3 (11.5)	0 (0.0)	3 (4.0)

ADA, Adalimumab; ETN, Etanercept; IFX, Infliximab; UST, Ustekinumab; SEC, Secukinumab; CVD, Cardiovascular Disease.

a Duration of psoriasis in years since symptom onset.

b Number of previous non-biological systemic treatments including phototherapy.

c Concomitant use with biological drugs.

Of the 106 courses started, 38 were discontinued. The most common cause of biological therapy discontinuation was loss of efficacy (N = 23, 60.5%), followed by discontinuation due to adverse events (n = 7, 18.4%) and interruption of medication supply due to government and bureaucratic issues (n = 5, 13.1%) (Table 2). Among the adverse effects, the most common was the development of infectious conditions (n  = 4, 57%).

**Table 2:** Reasons for discontinuing 106 courses of treatment with immunobiological drugs for psoriasis.

	Drug					
	Total number of courses	ADA	ETN	INF	UST	SEC
Loss of effectiveness, n (%)	23 (60.5)	11 (61.1)	6 (66.7)	3 (75.0)	3 (50.0)	0 (0.0)
Adverse effects, n (%)	7 (18.4)	4 (22.2)	1 (11.1)	1 (25.0)	1 (16.7)	0 (0.0)
Patient's decision, n (%)	2 (5.3)	1 (5.6)	1 (11.1)	0 (0.0)	0 (0.0)	0 (0.0)
Desire to get pregnant, n (%)	1 (2.6)	1 (5.6)	0 (0.0)	0 (0.0)	0 (0.0)	0 (0.0)
Interruption of drug supply, n (%)	5 (13.1)	1 (5.6)	1 (11.1)	0 (0.0)	2 (33.3)	1 (100)
Total number of discontinuations	38 (100)	18 (100)	9 (100)	4 (100)	6 (100)	1 (100)

ADA, Adalimumab; ETN, Etanercept; IFX, Infliximab; UST, Ustekinumab; SEC, Secukinumab.

The calculated median survival of immunobiological drugs (25/75 IQ) in months was 28.5 (8.0/52.0) for ADA, 23.0 (4.5/48.0) for ETN, 24.5 (9.0/52.0) for IFX, 19.0 (11.0/34.0) for UST and 12.0 (8.0/15.5) for SEC. The higher medians found for anti-TNFs reflect the longer time these drugs have been on the market. UST and SEC showed a lower median drug survival as expected, as they have been available on the market for a shorter time. However, they showed a considerably lower absolute number and percentage of treatment discontinuations, as observed in Table 2. Counting only the causes directly associated with the efficacy and tolerance in continuous treatment, anti-TNFs were responsible for 86.6% of discontinuations, versus 13.3% for UST and 0.0% for SEC.

The time until the interruption was longer for patients using UST compared to the group of patients who used anti-TNFs (Log Rank P test = 0.039) (Fig. 1). However, when UST was compared with each anti-TNF drug alone, there was a statistical difference only in the comparison between UST and ETN, with UST showing longer drug survival (log-rank p = 0.008). When interruptions for causes not directly associated with efficacy and tolerance in continuous treatment were censored, the results were similar, with UST again showing a longer time until the interruption in relation to the combined group of anti-TNFs (log-rank p = 0.037) and also concerning ETN alone (log-rank p = 0.018). However, this analysis also identified longer survival of UST compared to IFX (log-rank p = 0.045). Additionally, the analysis showed that SEC also had longer drug survival in relation to ETN (log-rank p = 0.047) and IFX alone (log-rank p = 0.042) (Fig. 1). A longer time to the interruption was also seen in the analysis of SEC with the combined group of anti-TNFs, although no statistical significance was observed (log-rank p = 0.090).

**Figure 1:**
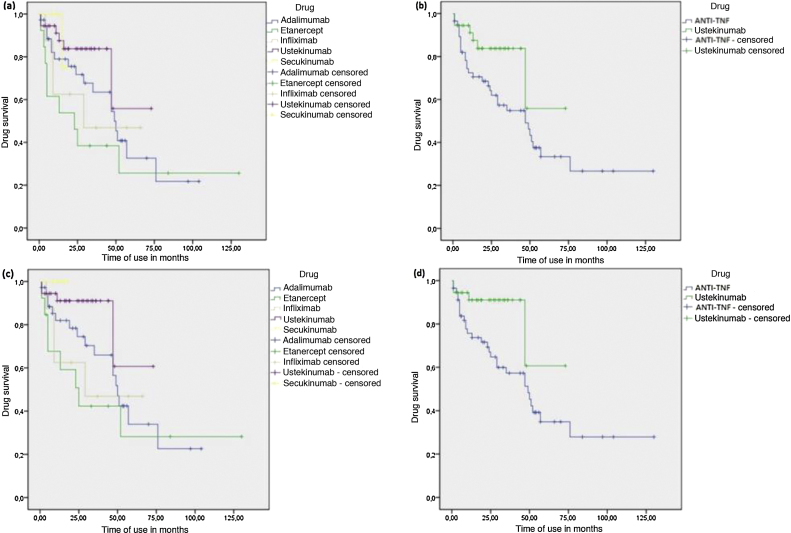
Kaplan-Meier - Drug survival analysis of biological agents in psoriasis. (A), Drug survival for each biological agent,considering all courses of treatment. (B), Drug survival comparing Ustekinumab (UST) and tumor necrosis factor inhibitors (anti-TNFs), considering all courses of treatment. (C), Drug survival for each biological agent, censoring interruptions for causes not directly associated with treatment efficacy and tolerance. (D), Drug survival comparing UST and anti-TNFs, censoring interruptions for causes not directly associated with treatment efficacy and tolerance.

Among the variables tested by Cox regression, the higher number of comorbidities (HR 1.735 95% CI 1.04-2.28, p = 0.032) and the previous use of a biological drug (HR 2.013, 95% CI, 1.03-2.87, p = 0.039) were statistically significant positive predictors for the likelihood of treatment interruption.

The results of the present study confirm previous observations of increased survival of immunobiological drugs in patients treated with UST and SEC.^1^, ^2^, ^3^, ^4^, ^5^, ^6^, ^7^, ^8^, ^9^ The lower rate of medication discontinuation in comparison to anti-TNFs is probably attributable to multiple factors, such as greater efficacy, favorable side effect profile, less frequent dose interval, and low immunogenicity.^2^, ^10^

As in other independent studies, the main cause of discontinuation during long-term treatment was the gradual loss of effectiveness. Adverse events (AE) were more frequent in the ADA group, corroborating what was seen in previous studies.^1^, ^2^, ^3^, ^4^, ^5^, ^6^, ^7^ And as in our study, infection development was also described by Gniadecki et al. as the most common AE.^6^

This study confirms the previous findings of increased survival of the anti-interleukin immunobiological drugs UST and SEC in comparison to anti-TNFs in the treatment of psoriasis. The presence of a higher number of comorbidities and previous exposure to a biological agent once again confirmed that they are negative predictors for maintaining the treatment.^3^, ^6^, ^7^ Studies with a larger number of patients and longer follow-up are necessary.

## Financial suport

None declared.

## Authors’ contributions

Elis Costa de Lima: Statistical analysis; study design and planning; drafting and editing of the manuscript; collection, analysis, and interpretation of data; critical review of the literature.

Juliana Catucci Boza: Approval of the final version of the manuscript; study design and planning; effective participation in research orientation; intellectual participation in the propaedeutic and/or therapeutic conduct of the studied cases; critical review of the manuscript.

Penélope Esther Palominos: Statistical analysis; effective participation in research orientation; critical review of the manuscript.

Ricardo Machado Xavier: Critical review of the manuscript.

Tania Ferreira Cestari: Intellectual participation in the propaedeutic and/or therapeutic conduct of the studied cases; critical review of the manuscript.

## Conflicts of interest

None declared.
